# Assessment of Prehospital Care for Pediatric Patients with Thermal Injuries: A Retrospective Study

**DOI:** 10.3390/jcm14124063

**Published:** 2025-06-09

**Authors:** Daniel Frank, Anna Forst, Christopher Ortmann, Stephan Gehring, Tatjana T. König, Eva Wittenmeier

**Affiliations:** 1 Department of Anesthesiology, University Medical Centre Mainz, Johannes Gutenberg University, 55131 Mainz, Germany; 2 Children’s Hospital, University Medical Centre Mainz, Johannes Gutenberg University, 55131 Mainz, Germany; 3 Department of Pediatric Surgery, Hannover Medical School, 30625 Hannover, Germany

**Keywords:** emergency medicine, TBSA-B, total body surface area burned, pediatric, anesthesiology, thermal injury

## Abstract

**Background/Objectives:** Accurate prehospital assessment of total body surface area burned (TBSA-B) is crucial for pediatric burn management, guiding resuscitation, fluid therapy, and transfer decisions. This study evaluates the accuracy of prehospital TBSA-B estimations compared to in-hospital expert assessment and examines their impact on prehospital management. **Methods:** This retrospective study analyzed 104 pediatric burn cases (median 17 months; 5 days–14 years) from 2017 to 2021. The primary endpoint was the difference between prehospital TBSA-B estimation and clinical measurement, with a clinically significant discrepancy defined as >5%. Secondary endpoints included the relationship between TBSA-B estimation and fluid therapy, analgesia, and hospital stay duration. **Results:** Prehospital TBSA-B estimations ranged from 2% to 40% (mean: 13.9%, SD = 4.4%) with scalds being the most common burn type (90.4%). Bland–Altman analysis showed a mean TBSA-B overestimation (bias) of 6.35%, with limits of agreement ranging from −6.97% (CI: −9.42 to −4.51) to 19.67% (CI: 17.21 to 22.12). No significant patterns in overestimation were associated with age, gender, or burn location. Fluid therapy volumes were independent of prehospital TBSA-B estimates, and analgesic administration varied by gender, with girls receiving less analgesia than boys, but showed no association with burn extent or severity. Hospital stay duration correlated proportionally with in-hospital assessed TBSA-B. **Conclusions:** Prehospital TBSA-B estimation was systematically overestimated, yet it did not influence fluid therapy decisions. Gender differences were observed in analgesic administration, while hospital stay duration was directly related to burn extent. These findings highlight the need for improved training and standardized tools to enhance prehospital burn assessment in pediatric patients.

## 1. Introduction

Thermal injuries are among the most frequent causes of trauma in childhood and represent a significant burden for both patients and healthcare systems. In Germany alone, more than 30,000 children under the age of 18 receive medical treatment for burn injuries each year, with approximately 6500 requiring inpatient care and over 2000 undergoing surgical procedures [1]. Timely and accurate prehospital assessment of burn severity is crucial for determining the appropriate level of care, initiating pain management, and deciding on fluid resuscitation and the need for transfer to a specialized burn center [2,3].

A variety of methods are available for estimating TBSA-B in the prehospital setting. These include the Rule of Nines by Wallace [4], which provides an age-independent surface area estimate; the Lund–Browder chart, which adjusts for anatomical proportions across age groups; and the Rule of Palms, where the patient’s palm represents approximately 1% of their body surface area [5]. While each method has strengths, several studies have identified considerable variability in TBSA-B estimation accuracy, particularly in pediatric populations, where differences in body proportions increase the risk of error [3,6].

Overestimation of TBSA-B in prehospital settings is a frequently reported phenomenon, especially in cases involving extensive burns or very young children [3]. Such inaccuracies can lead to unnecessary clinical interventions, including overtreatment with fluids or analgesics. Factors contributing to this overestimation may include inexperience of the first responders, difficulty applying existing estimation tools under time pressure, and the lack of pediatric-specific training [7].

Despite the clinical relevance of this parameter, the reliability of TBSA-B estimation by emergency physicians in pediatric patients remains poorly understood, and the downstream effects on other aspects of preclinical care—such as fluid resuscitation or analgesia—have not been sufficiently investigated [5]. Moreover, potential gender differences in prehospital treatment have rarely been explored. This retrospective study addresses these knowledge gaps by analyzing the accuracy of prehospital TBSA-B estimation in children with thermal injuries and comparing it with in-hospital expert assessment made upon hospital admission. Additionally, the study evaluates the relationship between TBSA-B estimation and prehospital fluid administration, analgesic and sedative regimens, and hospital length of stay. The findings are intended to inform future guidelines and training measures for pediatric burn care in emergency settings.

## 2. Materials and Methods

This retrospective cohort study analyzed pediatric burn cases (*n* = 104) treated at the University Medical Center Mainz between January 2017 and December 2021. Inclusion criteria comprised all pediatric patients (<18 years) with thermal injuries who were transported to the University Medical Center Mainz by emergency physician teams, provided that both prehospital documentation and hospital records were available for analysis. No explicit exclusion criteria were defined. During the study period, there were no cases with TBSA-B > 50% or prehospital intubation due to airway burns in the available dataset.

Sample size calculation and statistical methodology were guided by recommendations from the Institute of Medical Biostatistics, Epidemiology and Informatics (IMBEI), University of Mainz. The primary endpoint was defined as the mean absolute difference between the total body surface area burned (TBSA-B) estimated prehospital and the value determined clinically upon hospital admission. A clinically relevant difference was assumed to be ≥5%. With a standard deviation of ≤6% in both measurements, the standard deviation of the absolute difference was estimated at ≤8.5%. To ensure that the 95% confidence interval for the mean difference did not exceed 5% in width, at least 45 cases per gender group were required.

Data were extracted from prehospital emergency physician protocols and hospital records. Variables collected included patient demographics, burn cause and location, burn severity, initial vital signs, first aid measures, infusion volumes, medication administration, and transport modality. Pain assessment was documented using the Numeric Rating Scale (NRS) or Visual Analog Scale (VAS), where available. These scales were used to quantify the subjective pain intensity, typically on a 0 to 10 scale, and were noted if explicitly recorded in the emergency documentation. Hospital records were used to confirm clinical TBSA-B, burn classification, and treatment duration in intensive care and general wards. When TBSA-B was recorded as a range, the mean value was used for analysis. Burn locations were categorized into head, face, torso, and extremities; multiple locations were coded accordingly. Burn causes were classified as scalds, flame burns, contact injuries, or other. Burn severity grades were simplified to the highest degree recorded per case for consistency between prehospital and clinical documentation. For patients lacking weight data, the sex-specific 50th percentile value for age was used, based on standardized growth references.

Statistical analyses were conducted using IBM SPSS Statistics (Version 27.0.1.0). Descriptive statistics were computed for all variables. Associations between categorical variables were tested using Pearson’s chi-squared or Fisher’s exact test. Metric variables were assessed for normal distribution using histograms and Q-Q plots. Relationships between metric variables were examined using scatterplots and, where appropriate, linear correlation. Agreement between prehospital and clinical TBSA-B assessments was visualized using Bland–Altman plots and quantified via the intraclass correlation coefficient (ICC). Interrater reliability for burn severity grading was evaluated using weighted Cohen’s kappa. Depending on data characteristics, the *t*-test, Mann–Whitney U test, Wilcoxon signed-rank test, or Kruskal–Wallis test was applied. A two-sided significance level of 0.05 was used throughout.

The authors used a generative AI tool (ChatGPT, OpenAI, Version GPT-4) solely to enhance the language clarity and structure of the manuscript. The scientific content, data analysis, interpretations, and conclusions are entirely the work of the authors.

## 3. Results

A total of 104 pediatric patients with thermal injuries (*n* = 104) were included. Of these, 38 were female and 66 male. The children were predominantly infants and toddlers, with a median age of 17 months (range: 5 days to 14 years). The vast majority of injuries (82.7%) occurred in domestic or recreational settings. Scalds caused by hot liquids (e.g., water, tea, coffee) represented the most common mechanism of injury (90.4%), followed by flame burns (5.8%), contact injuries (1.9%), and other causes such as oil or fat (1.9%). Patient characteristics are shown in Table 1.

Vital signs were incompletely documented in the prehospital records. Oxygen saturation (SpO_2_) was recorded in 91 cases (median: 99%), heart rate in 90 (median: 135 bpm), respiratory rate in 63 (median: 25/min), and blood pressure in 26 (systolic) and 17 (diastolic) cases. The Glasgow Coma Scale (GCS) was noted in 89 cases (median: 15), and body temperature was measured prehospitally in 28 cases.

First aid by laypersons was documented in only 15 cases, including water cooling in 7 and other actions (e.g., clothing removal) in 5. Vascular access was established in 57 patients (52 intravenous, 5 intraosseous), while 31 received no access and 16 were undocumented. Notably, even among patients with estimated TBSA-B > 15%, vascular access was omitted in eight cases. The choice to place an intravenous or intraosseous line showed no statistically significant association with the extent of TBSA-B estimation on scene (Pearson χ^2^ = 1.87, *p* = 0.760; Fisher’s exact test = 1.587, *p* = 0.831). Wound coverage was reported in 30 cases; oxygen was administered in 14 patients, and 4 were intubated. Most patients (*n* = 83) breathed spontaneously in room air.

**Figure 1:**
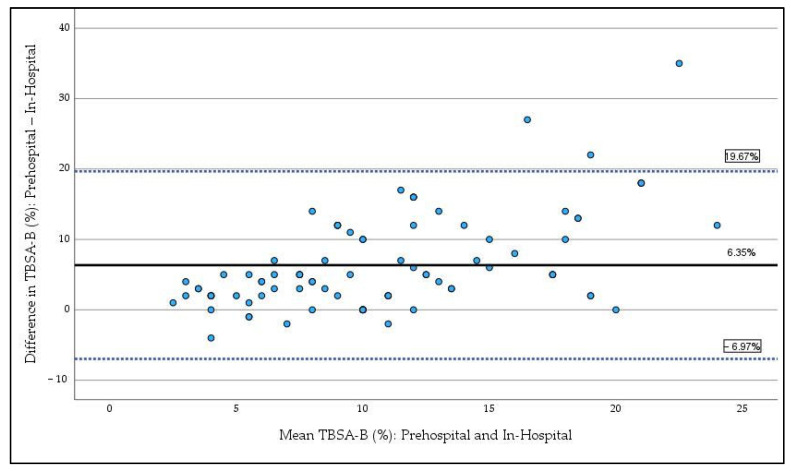
Bland–Altman plot comparing prehospital estimation and in-hospital expert estimation of TBSA-B. The solid line represents the mean difference (bias), while the dashed lines indicate the 95% limits of agreement. Data points show the difference between prehospital estimation and the mean of prehospital and in-hospital expert assessment of TBSA-B.

The prehospital estimation of TBSA-B demonstrated a statistically significant discrepancy compared to in-hospital expert assessment. Prehospital estimations ranged from 2% to 40% (mean: 13.9%, SD: 4.4%), whereas in-hospital TBSA-B values spanned 1% to 25% (mean: 8.0%, SD: 2.7%). Bland–Altman analysis (Figure 1) revealed a mean overestimation (bias) of 6.35% (95% CI: 4.91–7.78), with limits of agreement ranging from −6.97% (95% CI: −9.42 to −4.51) to 19.67% (95% CI: 17.21 to 22.12). Larger TBSA-B estimates were particularly prone to deviation, frequently exceeding the predefined limits of agreement. Reliability analysis using the ICC yielded a value of 0.223 (*p* = 0.119), classifying the agreement between prehospital and in-hospital assessments as poor.

No statistically significant patterns influencing the overestimation of TBSA-B were identified in relation to age, burn location, or gender. Regarding age, neither linear correlation analysis between overestimation and age nor comparisons of overestimation in infants versus older children (dichotomized age variable) revealed statistical significance (*p* = 0.123). Similarly, the analysis of burn location showed no statistically significant overestimation differences when comparing burns involving the head and face region to burns excluding these areas (*p* = 0.08). Lastly, comparisons between genders found no statistically significant differences in TBSA-B overestimation between boys and girls (*p* = 0.105).

The volume of prehospital fluid administration showed no significant correlation with prehospital TBSA-B estimation. The median infusion volume was 11.8 mL/kg (range: 0–55.6 mL/kg). Volumes did not differ significantly across TBSA-B groups (≤5%, 6–15%, >15%) (*p* = 0.685, Kruskal–Wallis test). In the subgroup of cases with documented body weight (*n* = 45), girls received a median volume of 20.8 mL/kg (range: 0–55.6 mL/kg), while boys received a median volume of 9.1 mL/kg (range: 0–55.6 mL/kg).

**Figure 2:**
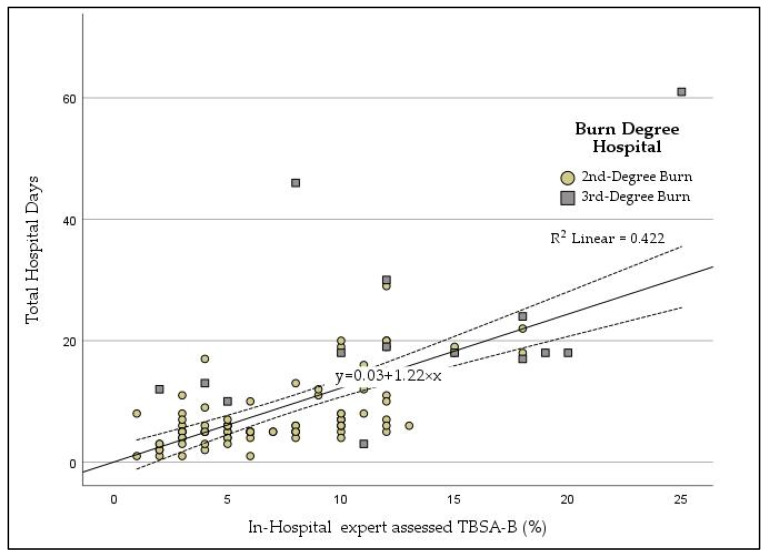
Scatter plot with regression line showing the total body surface area burned (TBSA-B, %, X-axis) and hospital length of stay in days. Circles represent second-degree burns; squares represent third-degree burns.

A positive linear correlation was observed between the in-hospital expert assessment of TBSA-B and the duration of hospital stay. The relationship can be expressed as: hospital stay (days) = 0.03 + 1.22 × TBSA-B (%), with a coefficient of determination (R^2^) of 0.422, indicating that 42.2% of the variability in treatment duration is explained by the extent of burns (Figure 2). Notably, the severity of burns did not exert an additional influence on hospital stay duration.

Pain levels were documented in 48% of cases studied (*n* = 104). The median pain score was 6/10 (range: 0–9) in girls and 7/10 (range: 0–10) in boys. Analgesic administration was documented in 80% of cases studied. Ketamine combined with midazolam was the most frequently used regimen for pain management and sedation (30.2%), followed by the combination of an opioid with other substances (propofol or dimenhydrinate, 11.5%), or opioid monotherapy (10.4%). Gender-based differences were noted in the choice of analgesics even though the estimated TBSA-B was similar with a median of 12% for boys and girls. Girls were more likely than boys to receive only weak analgesics, such as Paracetamol or Ibuprofen (14.7% vs. 3.2%), sedatives alone (8.8% vs. 4.8%), or no medication at all (14.7% vs. 3.2%). Boys more frequently received ketamine-based regimens (64.5% vs. 35.3%).

**Figure 3:**
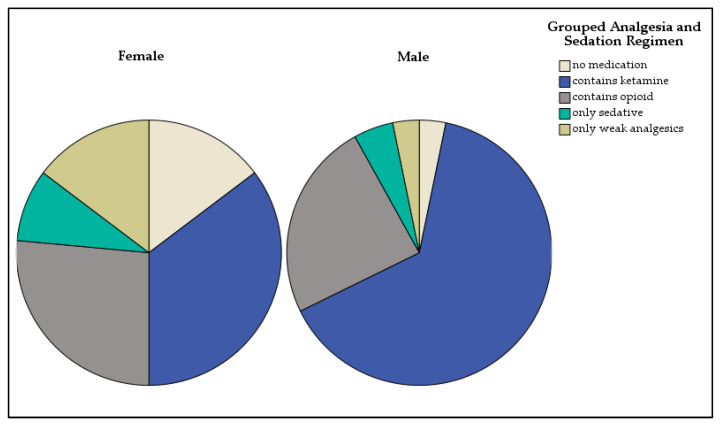
Distribution of analgesia and sedation regimens in girls and boys.

For burns with a TBSA-B > 15%, no medication was administered in three cases, and two cases involved only weak analgesics. Analgesia and sedation regimens are shown in Figure 3. No statistically significant association between the chosen analgesic–sedative regimen and TBSA-B estimated was identified.

To further investigate the observed gender difference in the administration of strong analgesics, a logistic regression model was calculated. The dependent variable was the administration of a strong analgesic regimen (e.g., ketamine-based), with gender (male vs. female) as the primary independent variable. Burn severity and total body surface area burned (TBSA-B) were included as covariates to control for potential confounding factors. The analysis showed that male patients had significantly higher odds of receiving strong analgesia compared to female patients (odds ratio [OR] = 1.67; 95% confidence interval: 1.7–16.4; *p* = 0.0038). Neither burn severity (*p* = 0.3687) nor TBSA-B extent (*p* = 0.0897) were statistically significant predictors in the model.

Among the 104 cases, ambulances were used for transport in 63 cases, while air ambulances (helicopters) were employed in 41 cases. In total, 96 transports were conducted as primary transfers, and 8 were secondary transfers. Analysis of transport modes across TBSA-B categories showed that even children with ≤5% TBSA-B were transported via helicopter in nine cases. The choice of transport mode was found to be independent of burn severity or extent.

Documentation of alarm times was available for 64 cases, while arrival times at the hospital were recorded for 56 cases. In 45 instances, the interval between alarm activation and hospital arrival could be calculated, with an average duration of 1.05 h (range: 23 min to 2.1 h).

## 4. Discussion

This study showed that prehospital estimation of TBSA-B demonstrated a significant and systematic overestimation compared to in-hospital expert assessment, with a mean discrepancy of 6.35% and limited reliability with limits of agreement exceeding 25%, especially in larger burns in a large cohort of infants, children and adolescents. These findings highlight the challenges faced by emergency medical personnel in accurately estimating TBSA-B during initial evaluations.

Prehospital overestimation of TBSA-B in our study are supported by earlier research suggesting an overestimation of TBSA-B by emergency in children [2,3]. One study involving only 27 children did not demonstrate a clinically relevant overestimation in the Bland–Altman analysis; however, it similarly observed a decline in agreement between emergency medical personnel and burn specialists with increasing burn extent [2]. McCulloh et al. reported a mean overestimation of total body surface area burned (TBSA-B) by 8.1% in prehospital settings, based on data from 67 pediatric patients, with a mean clinically determined TBSA-B of 18.9%, in contrast to 8% in our cohort [3]. Therefore, in our study, overestimation is much more pronounced in relation to the actual burn extent. Factors contributing to this overestimation may include limited clinical experience with pediatric burn injuries, difficulty applying standard estimation tools under time constraints, and the unique anatomical proportions of children, particularly in younger age groups [8].

In this study, no significant associations were found between the prehospital overestimation of TBSA-B and potential influencing factors, such as age, burn location, or gender. Similarly, no significant differences were observed between burns involving the head and face region versus other areas, or between boys and girls. McCulloh et al. noted significant misjudgments in children below five years of age with larger burn areas [3]. Such discrepancies can be attributed to factors such as the complexity of assessing burn areas in children, varying levels of experience among first responders, and limitations in available tools [3,9].

Furthermore, a slight tendency for excess administration of fluids was observed, with a median administered volume of 11.8 mL/kg/h, exceeding the recommended upper limit of 10 mL/kg/h. However, there was no significant correlation with prehospital TBSA-B estimations. This indicates that the volume of fluid therapy administered prehospital was not guided by the estimated extent of burns, highlighting a uniform approach to infusion volume irrespective of the burn extent assessed. In contrast to our findings, previous studies observe a tendency for access infusion in prehospital care, which frequently attribute excessive fluid administration to overestimation of TBSA-B [5,10]. Goverman et al. reported unnecessary fluid therapy in 86% of pediatric burn cases with minor injuries, with overhydration occurring in 59% of patients regardless of gender [5]. This practice may stem from concerns about hypovolemic shock in children, as guidelines emphasize the risk of shock in burns involving ≥10% TBSA-B [11]. However, evidence suggests that such concerns are generally unwarranted during the short prehospital phase, as hypovolemic shock typically develops after one to two hours in burns exceeding 10% TBSA-B, with peak edema formation occurring 8–12 h post-injury [11]. Adams and Vogt argue that preemptive fluid therapy is unnecessary in most prehospital scenarios and that early signs of shock are more likely attributable to severe concomitant injuries rather than burn-related hypovolemia [11]. Notably, this study found no documented evidence of concomitant injuries or signs of shock in the analyzed cases. These findings underscore the need to align prehospital fluid therapy with evidence-based guidelines to prevent potential complications from excess intravenous fluid administration such as pulmonary edema [7] and increased fluid creep and tissue swelling, thereby delaying wound healing and increasing the need for skin grafting [12].

In this study TBSA-B, assessed by in-hospital experts and hospital stay duration, correlated with 42.2% of the variability in treatment duration explained by burn extent, while no additional influence of burn severity was identified (Figure 2). The strong correlation between TBSA-B and hospital stay duration underscores the significance of accurate TBSA-B assessment for predicting resource utilization and planning treatment strategies. This finding aligns with a prior single center retrospective cohort study done by Gale et al. including 761 patients that emphasizes the proportional relationship between burn size and hospitalization duration, as larger burns often require extended monitoring and intervention [13]. Interestingly, the absence of a significant influence of burn severity on hospital stay duration in our study suggests that the extent of burns, rather than their depth, primarily dictates treatment demands in our cohort. However, our cohort largely consisted of scald injuries, which typically result in superficial partial thickness burns [14].

Boys more often received ketamine in this study, while girls more frequently received weak analgesics or no medication. As noted in the literature, these weak analgesics are considered insufficient for the initial care of thermally injured children [15]. Surprisingly, neither burn extent nor burn severity did significantly influence analgesic choice. The observed gender differences in analgesic and sedative administration suggest a potential bias in prehospital pain management strategies. To date no comparable study could find this potential gender difference in analgesics. This discrepancy may partially be explained by clinical findings of more frequent third-degree burns in boys compared to girls, potentially influencing first responders’ perception of pain severity even though third-degree burns are in reality less painful due to the injury of pain receptors. However, the absence of a significant association between burn extent and analgesic choice highlights variability in prehospital practices, likely driven by subjective assessments rather than standardized protocols. Effective early pain control is crucial for long-term outcomes and patient compliance, resulting in improved wound healing and functional outcomes [16] and prevention of posttraumatic stress disorder [17]. While this finding reflects the pattern within our investigated cohort, it should be interpreted as a descriptive observation. It highlights the need for further prospective research to explore potential gender disparities in prehospital pain management and their underlying causes.

This study has several limitations that must be considered when interpreting the findings. The prehospital emergency physician protocols varied in structure across different emergency services, and in some cases, burn injuries were not explicitly documented, requiring interpretation of free-text entries. Documentation was sometimes incomplete or handwritten and difficult to read, leading to missing or unreliable data for certain variables such as first aid measures, cooling, thermal protection, and vital signs. Additionally, the years 2020 and 2021—part of the study period—were marked by the COVID-19 pandemic. The extreme conditions in some phases of the pandemic may have influenced both clinical practices and documentation quality, potentially affecting the results.

## 5. Conclusions

Emergency treatment of pediatric burns largely influences patient compliance and long-term outcomes, highlighting the crucial role of first responders and prehospital emergency care in pediatric burn patients. Addressing the observed overestimation of TBSA-B could improve prehospital care significantly. In adults, evidence already supports implementing enhanced training programs for emergency medical personnel, using software-based tools for TBSA-B calculation [18,19] or leveraging telemedicine to enable real-time consultations with burn specialists [5,8]. These tools could also be tested and implemented in pediatric patients. Further studies should be conducted to evaluate if these strategies reduce errors in TBSA-B estimation but also optimize clinical decision-making, including fluid resuscitation and analgesia in pediatric patients. These findings support targeted improvements in training and documentation, and may inform future guidelines for prehospital pediatric burn care.

## Figures and Tables

**Table 1:** Characteristics of assessed pediatric patients with thermal injuries. Values are median (min-max) or absolute numbers (proportion), * numeric rating scale.

Age	17 Months (5 d–14 y)
Sex	
Male	66 (63%)
Female	38 (37%)
Burn Types	
Scald	94 (90.4%)
Flame	6 (5.8%)
Contact	2 (1.9%)
Others	2 (1.9%)
Burn Location	
Head	6 (5.8%)
Face	32 (30.8%)
Trunk	70 (67.3%)
Extremities	78 (75%)
Pain score (NRS *)	
Male	7/10 (0–10)
Female	6/10 (0–9)
Type of Transport	
Ambulance	63 (61%)
Air ambulance	41 (39%)

## Data Availability

The data supporting the findings of this study are not publicly available due to ethical and legal restrictions. As the study is based on retrospective patient data collected without individual consent, sharing the dataset is not permitted in accordance with data protection regulations. Parts of the data of the manuscript are parts of the doctoral thesis of A.F.

## Abbreviations

The following abbreviations are used in this manuscript:

TBSA-B	Total body surface area burned
NRS	Numeric rating scale
ICC	Intraclass correlation coefficients
